# Supplementary material for: Exogenous Nitric Oxide Promotes the Growth and Cadmium Accumulation of Alfalfa (Medicago sativa) Seedlings Under Cadmium Stress
Source: Plants (Basel). 2025 Oct 25;14(21):3264. doi: 10.3390/plants14213264 (PMC12608502; doi:10.3390/plants14213264)
Supplement: Supplementary file 1 [file plants-14-03264-s001.zip › plants-3823715-supplementary.pdf]

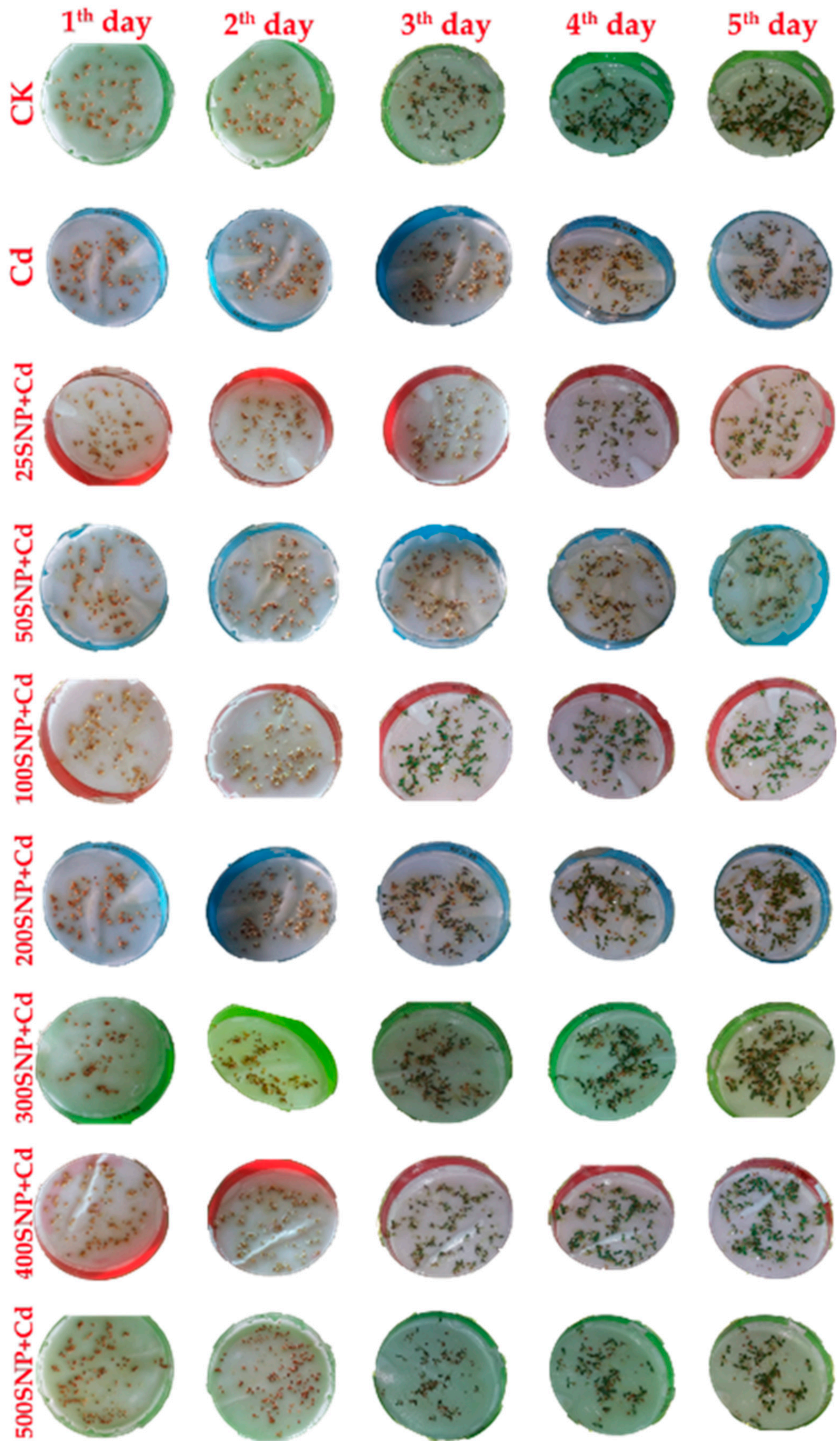

Figure S1. The growth conditions of all the seedlings treated with different concentration of SNP under 30 μM Cd stress.
